# MixImages: An Urban Perception AI Method Based on Polarization Multimodalities

**DOI:** 10.3390/s24154893

**Published:** 2024-07-28

**Authors:** Yan Mo, Wanting Zhou, Wei Chen

**Affiliations:** 1 School of Information Engineering, Nanchang Hangkong University, Nanchang 330063, China; 2304085401015@stu.nchu.edu.cn; 2 College of Aeronautics Engineering, Nanjing University of Aeronautics and Astronautics, Nanjing 210016, China; 3 College of Geoscience and Surveying Engineering, China University of Mining & Technology, Beijing 100083, China; chenw@cumtb.edu.cn

**Keywords:** urban perception, deep learning, semantic segmentation, polarization

## Abstract

Intelligent urban perception is one of the hot topics. Most previous urban perception models based on semantic segmentation mainly used RGB images as unimodal inputs. However, in natural urban scenes, the interplay of light and shadow often leads to confused RGB features, which diminish the model’s perception ability. Multimodal polarization data encompass information dimensions beyond RGB, which can enhance the representation of shadow regions, serving as additional data for assistance. Additionally, in recent years, transformers have achieved outstanding performance in visual tasks, and their large, effective receptive field can provide more discriminative cues for shadow regions. For these reasons, this study proposes a novel semantic segmentation model called MixImages, which can combine polarization data for pixel-level perception. We conducted comprehensive experiments on a polarization dataset of urban scenes. The results showed that the proposed MixImages can achieve an accuracy advantage of 3.43% over the control group model using only RGB images in the unimodal benchmark while gaining a performance improvement of 4.29% in the multimodal benchmark. Additionally, to provide a reference for specific downstream tasks, we also tested the impact of different combinations of polarization types on the overall segmentation accuracy. The proposed MixImages can be a new option for conducting urban scene perception tasks.

## 1. Introduction

With the acceleration of urbanization, cities have become a hub of human activities, making urban perception a focal point. Urban perception refers to the use of sensors and data analytics technologies to comprehend urban elements, offering crucial upstream support for tasks such as autonomous driving [1,2], building identification [3,4], pedestrian detection [5,6], and analysis of environmental sustainability [7,8]. In recent years, driven by deep learning, urban perception has been advancing toward intelligence. Semantic segmentation is one of the most advanced urban perception methods, enabling the understanding and analysis of urban information at the pixel level [9]. At present, several pioneers have proposed many effective semantic segmentation models [10,11,12,13,14,15], significantly pushing the performance boundaries for a wide range of remote sensing vision tasks.

The aforementioned methods solely utilize unimodal RGB images for semantic segmentation, as they encapsulate rich visual information. However, in natural urban scenes, the occlusion of objects by lighting can create strong shadow regions, and the visualization effect of the real shape or texture of ground objects is reduced [16]. Since the unimodal RGB image lacks the information dimension required to fully perceive the unconstrained environment, it needs to be supplemented with the information of other data types [17]. Polarization data contain information about the oscillation direction and intensity of light waves, and they are widely used in dehazing, deshadowing, and other applications. The polarization characteristics of light are related to different surface materials, roughness, geometric structures, etc. Therefore, polarization data can provide some imperceptible features distinct from RGB images [18], making them an excellent source of high-quality data for enhancing urban perception. In addition, different from models based on convolutional neural networks (CNNs), the large effective receptive field of vision transformers (ViTs) [19,20,21] can provide support for precise discrimination of shadow regions. Although ViTs are efficient, the characteristics of its core operator, self-attention, hinder the performance of pure ViT-based structures in perceiving fine-grained semantics, especially for multimodal data with significant differences in information entropy.

Inspired by the aforementioned factors, this study proposes a novel semantic segmentation model called Miximages, which can utilize multimodal polarization data for urban perception. Specifically, MixImages first refines the features of input RGB-P data through a carefully designed multimodal fusion module. Subsequently, we design a CNN-Transformer mixture backbone to obtain discriminative cues for urban shadow regions from a global perspective while enhancing fine-grained representations. Finally, coupling the two parts with a semantic segmentation decoder to form our MixImages model. Furthermore, for more precise segmentation, we introduce a decoding head before the pixel-level classifier to optimize multimodal features.

To test the performance of our model, we selected some state-of-the-art semantic segmentation models, including unimodal and multimodal approaches, from the past few years to conduct comprehensive experiments on a polarization dataset. The results show that our MixImages can achieve competitive performance in each set of experiments. Our approach achieved accuracy improvements of 3.43% and 4.29% over the best-performing compared models in unimodal and multimodal benchmarks, respectively. Notably, we explored how different combinations of polarization states affect the model’s performance to provide references for specific downstream tasks. Specifically, the main contributions of this paper are as follows:

(1) We construct a CNN–transformer mixture network backbone suitable for urban perception using RGB-P multimodal data combinations.
(2) We propose a novel semantic segmentation model, MixImages, which can surpass some state-of-the-art methods on a polarization dataset of urban scenes.
(3) We explore how different types of polarization data combinations affect the model’s performance, providing references for specific downstream tasks.

The rest of the paper proceeds as follows: Section 2 describes the state of related work, Section 3 elaborates on the model proposed in this study, Section 4 presents the experimental setup and results, and Section 5 reveals the conclusions of this research.

## 2. Related Works

### 2.1. CNN-Based Models

After large-scale public datasets and computing resources became available, CNNs began to become the benchmark model for computer vision (CV). The advent of AlexNet [22] has driven the deep learning model to a deeper level. A series of deeper and more effective models, such as VGG [23], GooleNet [24], ResNet [25], DenseNet [26], and EfficientNet [27], have been proposed, further promoting the wave of deep learning. The most popular of these is ResNet, which includes a residual connection that alleviates the problem of gradient disappearance and gradient explosion in deep networks and provides a theoretical basis for deeper networks. In addition to changing the design of the architecture, some studies focus on introducing more complex convolution operators, e.g., depthwise separable convolution [28,29,30], deformable convolution [31,32], and a series of convolution-based attention mechanisms for channel dimension and spatial dimension [33,34,35,36]. With the significant success of transformers in the field of natural language processing, ViTs have also swept the field of image processing [37,38,39] and are becoming the first choice for large-scale visual models [13]. Considering that the success of ViTs stems from its large and efficient receptive field, modern CNNs are trying to find better components to improve their performance on visual tasks. Some works introduced improved convolutions with long-distance dependencies [11] or dynamic weights [40], making meaningful attempts to demonstrate the competitiveness of CNNs in the era of large parameters. Other works directly replaced some or all layers in classic CNNs with transformer blocks [41]. To speed up optimization, the self-attention of these works is computed within local windows, and although they achieve better accuracy than the original model, their expensive memory accesses lead to significantly higher actual latency than CNNs. Moreover, recent research has also attempted to expand the receptive field of CNNs by super-large dense kernels (e.g., 31 × 31) [42], but there is still a certain gap compared with state-of-the-art ViTs.

### 2.2. Transformer-Based Models

Benefiting from the multi-head self-attention (MHSA) mechanism and dynamic adaptive spatial aggregation, ViTs can learn more robust and richer visual representations than CNNs. For this reason, new vision models mostly tend to use transformers. Among them, the ViT [43] is the most representative model. As the visual backbone of a pure transformer structure, it creatively incorporates the concept of treating images as continuous patches and improves the performance of image classification tasks. However, because of its low-resolution feature maps, quadratic computational complexity, and lack of image-sensitive hierarchical structures, it is unsuitable for downstream tasks that require advanced attributes of images. Some works have applied the ViT to intensive vision tasks, such as object detection and semantic segmentation, via direct upsampling or deconvolution, but the performance was relatively low. To this end, PVT [19,38] and Linformer [44] performed global attention on the maps of downsampled keys and values; DAT [45] employed deformable attention to sparsely sample information from value maps, while Swin transformer [46] and HaloNet [47] developed local attention mechanisms and used shift windows to complete the information interaction among adjacent local regions. In addition, some works have noticed that ViTs lack the inductive bias of CNNs and the ability to model local details. By introducing a convolution operator based on the transformer architecture, the upper limit of the performance of the model is further improved.

### 2.3. Semantic Segmentation Methods

Semantic segmentation is one of the most advanced urban perception methods. FCN is a seminal work of semantic segmentation [48] and is the first CNN structure to effectively solve semantic segmentation tasks in an end-to-end form. Since the introduction of FCN, methods based on CNNs have dominated semantic segmentation. Another groundbreaking work is SegNet [49], which involves a standard encoder–decoder architecture for semantic segmentation. Inspired by the above works, researchers have developed techniques such as long skip connections, context dependence, multi-scale strategies, etc., for semantic segmentation. Among them, UNet [50] developed a long skip connection between the encoder and the decoder based on the ResNet residual structure, which ingeniously bridged the semantic gap between the encoding end and the decoding end and became the most commonly used semantic segmentation model for a period of time. After that, based on the U-shaped structure of UNet, a series of UNet-like network models appeared, e.g., UNet++ [51] combined with dense connections, Attention UNet [33] with context dependence, and UNet3+ [52], which introduces multi-scale. Until the popularity of the transformer emerged, the variant UNet with the transformer block was still a hot focus of researchers and showed competitive performance [53,54]. In addition, the DeepLab series of networks [55,56] shone brilliantly during the same period as UNet. As representative works of multi-scale strategies, they introduce dilated convolutions to fuse multi-scale features. Different from the early semantic segmentation models, in the field of computer vision over the past few years, semantic segmentation models are no longer separated out as specialized visual task models. The latest state-of-the-art semantic segmentation algorithm is actually derived from the effective combination of the aforementioned transformer-based and CNN-based visual basic model and the encoder. In other words, the general visual backbone with a multi-level architecture and the neck with a pyramidal hierarchical structure are connected at the same resolution level to form an encoder–decoder structure as a whole [13,15]. In addition, for complex scenes that are difficult to handle with unimodal data, researchers have proposed a series of emerging multimodal semantic segmentation tasks named RGB-P, RGB-T, and RGB-D with the help of other modal data sources, such as polarization, thermal infrared, and depth [57,58,59]. However, most of the above methods are based on CNN approaches, and there is limited research that combines them with ViTs. Therefore, in this work, we utilize polarization as the additional modality and construct a mixture urban perception model that combines CNN with the ViT.

## 3. Methodology

**Figure 1:**
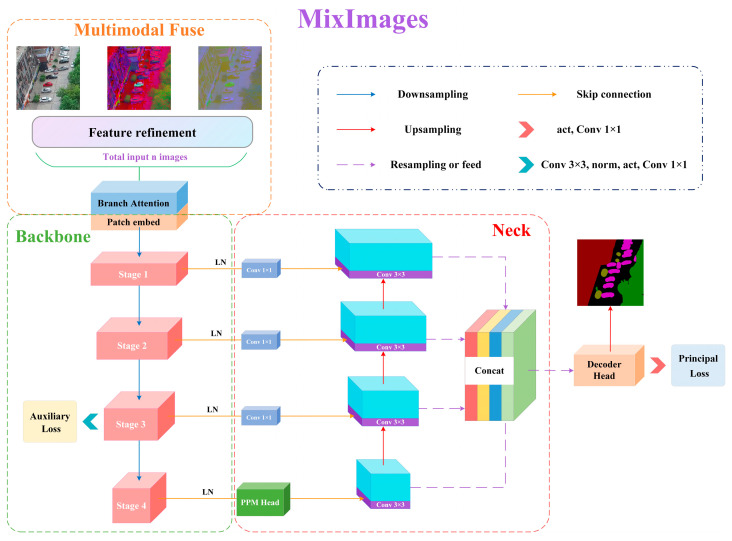
Overall structure diagram of the proposed MixImages.

As shown in Figure 1, the proposed MixImages follows the standard encoder–decoder structure, and it is coupled with a multimodal fuse, backbone, and neck modules, ultimately outputting segmented probability distribution results.

### 3.1. Multimodal Fuse Module

Traditional semantic segmentation models usually have only one input interface, which means the model considers only one modality of input. In this study, we design a multimodal fusion module to enable MixImages to accept multiple inputs. Specifically, the multimodal fuse module includes n congruent feature refinement structures and a modal attention block. Each feature refinement structure starts with multiple parallel input interfaces and then maps each feature map to a higher order to refine the features. Considering this part is based on fine-grained operations, to save computational efficiency, we only stack a few convolutional kernels continuously and couple them through residual connections. Based on the above operations, the input source data x_i_ ℝ^B×Ci×H×W^ is mapped to x_i′_∈ℝ^B×C×H×W^(C_i_ < C) to obtain a richer feature representation, where B represents batch size, *C* represents channels, i refers to the i-th modal, *H* and *W* denote the height and width of the image, respectively. Here, we employ a combination of 3 × 3 convolutions and a rectified linear unit to achieve dimensionality expansion to construct high-dimensional non-linear features. Then, the results of feature refinement structures are adaptively fused through the modal attention block.

There are two ways to implement modal attention: channel attention and element-wise sum. For channel attention, the multimodal feature map stacks from each modal are concatenated in the channel dimension to let the network automatically select the features of more important channels. Squeeze-and-excitation (SE) [36] and the convolutional block attention module (CBAM) [34] are two widely used methods; theoretically, any plug-and-play channel attention mechanism can serve as an equivalent replacement role. The element-wise sum is a lightweight branch attention fusion method developed in this study. Specifically, we first initialize a list with a length equal to the number of the high-order feature maps (float32 format) and assign each member of the list gradient attributes. Then, these dynamic parameters with gradient backpropagation capability are sequentially assigned as coefficients to each feature map to perform the weighting sum operation. In this way, the network can adaptively assign weights to each modal during the training process. To keep the fused data distribution unchanged and make the training more stable, we perform a Softmax operation on all coefficients before weighting to ensure that the sum of the weights of each modal is 1. The calculation process can be expressed as

(1) $$\begin{array}{c} {\mathrm{MF}}_{\mathrm{out}}=\mathrm{Attention}\left({\left[{\mathrm{FR}}_{i}\left(x_{i}\right)\right]}_{i=1}^{n}\right)\\ \mathrm{Attention}=\mathrm{Softmax}\left(\overset{n}{\underset{i=1}{\sum }}a_{i}x_{i}|\dim=1\right),\;\;\;x_{i}\in R^{B\times C\times H\times W} \end{array}$$

where $x_{i}$ denotes the input of the i-th modal data; $a_{i}$ represents the i-th coefficient with gradient backpropagation capability; ${\mathrm{FR}}_{i}\left(\cdot \right)$ represents the feature refinement structure of the i-th branch; and ${\left[\cdot \right]}_{i\;=1}^{n}$ represents a set containing n elements.

### 3.2. Backbone

We start by constructing a CNN–transformer mixed basic block to carry our network backbone and devising the corresponding stacking principles so that it can be expanded to different scales like state-of-the-art methods.

#### 3.2.1. Operator

**CNN vs. transformer.** The way to choose the operator depends on the angle from which the image is interpreted. CNNs treat images as 2D feature maps, while ViTs treat them as 1D tokens. The difference between them is as follows:

(1) Local information or long-distance dependence. The 2D structure of the standard regular convolution determines that the range of its perception of features is the pixel in the center of the filter and its neighborhood. This kind of operator is more sensitive to the context relationship between local pixels, which is beneficial to extracting the texture details of small target objects in RS images. Unfortunately, it is not very good at grasping global information. The expansion of the receptive field in CNNs depends on the continuous deepening of the network depth, while even very deep CNNs still cannot obtain the same effective receptive field as ViTs. On the contrary, for ViTs, since the data format received by the transformer is a 1D sequence, in the early stage of the network, the 2D images are divided into n equally sized patches and transformed into a 1D token form. Obviously, the local relationship between adjacent pixels is greatly weakened in this process. On the other hand, ViTs have an unconstrained perception dimension and can model long-distance dependencies well, which is more conducive to the positioning of ground objects and the segmentation of large target objects.
(2) Static spatial aggregation or dynamic spatial aggregation. Compared with self-attention, which has the ability of adaptive spatial aggregation, standard regularized convolution is an operator with static weights. It has strong inductive biases, such as translation equivalence, neighborhood structure, two-dimensional locality, etc., which allow CNNs to have faster convergence speed than ViTs and require less data, but they also limit the ability to learn more general and more robust feature patterns.

#### 3.2.2. Basic Block

Existing work shows that ViTs with larger effective receptive fields usually perform better than CNNs in downstream vision tasks driven by massive data. However, the cost of real data acquisition and preprocessing is expensive. Furthermore, considering the complementarity of the two operators, we construct a mixed basic block that consists of a global perception unit based on self-attention and a local perception unit based on convolution.

**Figure 2:**
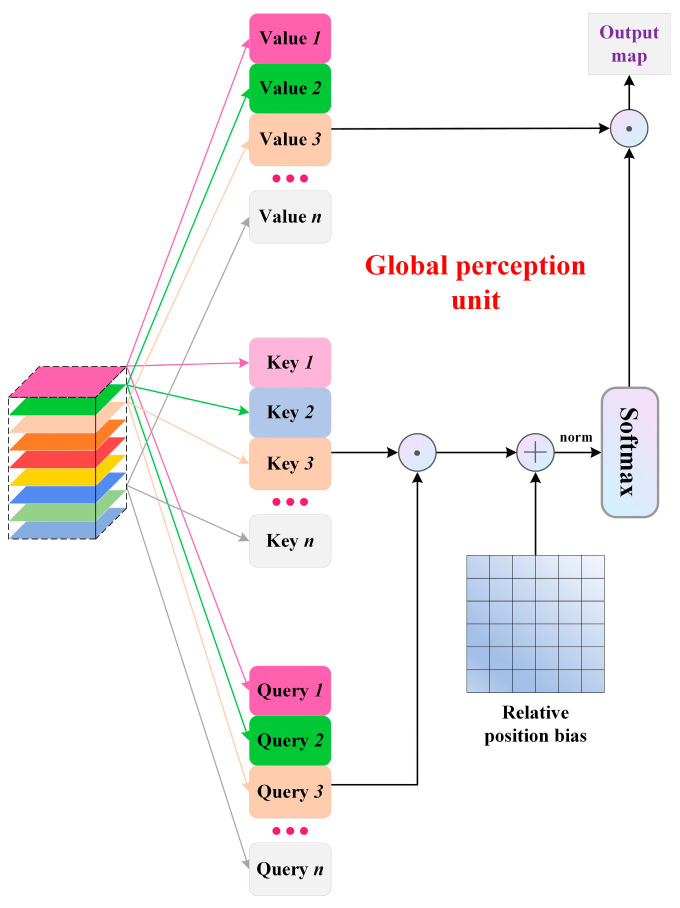
Overall calculation process of global perception unit.

In the global perception unit, although optical information varies among different polarization states, their spatial geometric information remains consistent. Therefore, we employ multimodal self-attention to learn these rich spatial features. We first replicate the ensemble of multimodal feature maps threefold through convolution and then flatten them into 1D tokens. Subsequently, we compute vector self-attention correlation representations between modal tokens. Considering that the computational complexity of self-attention is quadratic with respect to the image size, we deploy self-attention based on non-overlapping local windows. Within each window, for any feature matrix x composed of n tokens, the self-attention calculation process is

(2) $$\begin{array}{c} \mathrm{Self}-\mathrm{Attention}=\mathrm{Softmax}\left(\frac{{\mathrm{qk}}^{T}}{\sqrt{\dim}}+b\right)v \end{array}$$

where q (Query), k (Key), and v (Value) are the results of x after convolution and b represents the relative position bias. In the n × n attention map obtained by $q\cdot k^{T}$, the value of the i-th row and j-column (i, j < n) is the attention assigned to the j-th token when paying attention to the i-th token. After adding relative position information b to it, the attention map normalized by Softmax(·) is multiplied by v to obtain the feature matrix after self-attention weighted aggregation. In the actual process, the MHSA is used, and each head outputs a subspace of the sequence. Thus, in the end, the feature matrices obtained by different heads need to be fused. The entire process is illustrated in Figure 2. While performing self-attention in local windows is effective, the lack of interaction across windows hinders feature learning. To this end, we construct a shifted window inspired by the Swin transformer; more details are in [46].

For the local perception unit, considering that convolution can improve the lack of local information in self-attention, we use a convolution-based inverted residual structure to replace the MLP-based feedforward (FFD) block in traditional ViTs to form our local perception unit. Its calculation process can be represented as

(3) $$\begin{array}{c} \hat{x}=x_{\mathrm{SA}}+{\mathrm{DS}}_{3\times 3}\left(\mathrm{ReLU}\left({\mathrm{Conv}}_{1\times 1}\left(x_{\mathrm{SA}}\right)\right)\right) \end{array}$$

where $x_{\mathrm{SA}}$ and $\hat{x}$ represent the results after the self-attention operation and the output of the local perception unit, respectively; ${\mathrm{Conv}}_{k\;\times \;k}$ represents k × k (k = 1 here) convolution; ${\mathrm{DS}}_{3\times 3}$ represents k × k (k = 3) depthwise separable convolution (depthwise convolution + pointwise convolution). With the aforementioned operations, we can efficiently model local relationships.

#### 3.2.3. Downscaling Rules

To obtain multi-scale feature maps, we reduce the resolution of the feature maps to $\frac{1}{4}$ of the previous one through patch embedding. Then, each downsampling layer reduces the resolution to $\frac{1}{2}$ of the previous one, and the number of channels changes accordingly. Patch embedding divides the feature map (ℝ^B×C1×H×W^) into n patches 4 × 4 in size and stretches it into a 1D token form. This process is accomplished by a standard regular convolution with a kernel size of 4 × 4 and a stride of 4, outputting a feature map of x ∈ ℝ^B×C2×(H/4)×(W/4)^.

#### 3.2.4. Stacking Rules

In this part, we first enumerate the relevant hyperparameters of the MixImages’s backbone as follows:

C*_i_*—the number of channels in the i-th stage;

D*_i_*—the number of basic blocks in the i-th stage;

H_i_—the number of heads in the i-th stage;

G_i_—the number of groups in the i-th stage.

Since our backbone has 4 stages, a variant is determined by 16 hyperparameters, and the search space is too large. Referring to the existing technology [11,13,15], we summarize the stacking principle into the following three points: (1) C_i_ = 2^i−1^C_1_. The number of channels of the last three stages is expanded to twice that of the previous ones. (2) D_1_ = D_2_ = D_4_, D_3_ > D_1_. Use the same number of basic blocks for the 1st, 2nd, and 4th stages and less than the number of those in the 3rd stage. (3) H_i_ = C_i_/H′, G_i_ = C_i_/G′, H′, and G′ denote the dimensions of each head and each group, respectively. Use the same dimension for headers and groups in each stage, and the number of corresponding headers and groups is determined by the number of channels corresponding to the stage. Based on the above stacking rules, we construct hierarchical backbones with different capacities, as shown in Table 1.

### 3.3. Decoder Neck

Drawing on the decoder of the leading semantic segmentation algorithm, we use UperNet [60] as the decoding neck of MixImages, which is composed of a PPM head [61] and FPN [62] in series. As the neck part does not belong to the contribution of this article, it will not be further detailed.

### 3.4. Decoder Head

**Figure 3:**
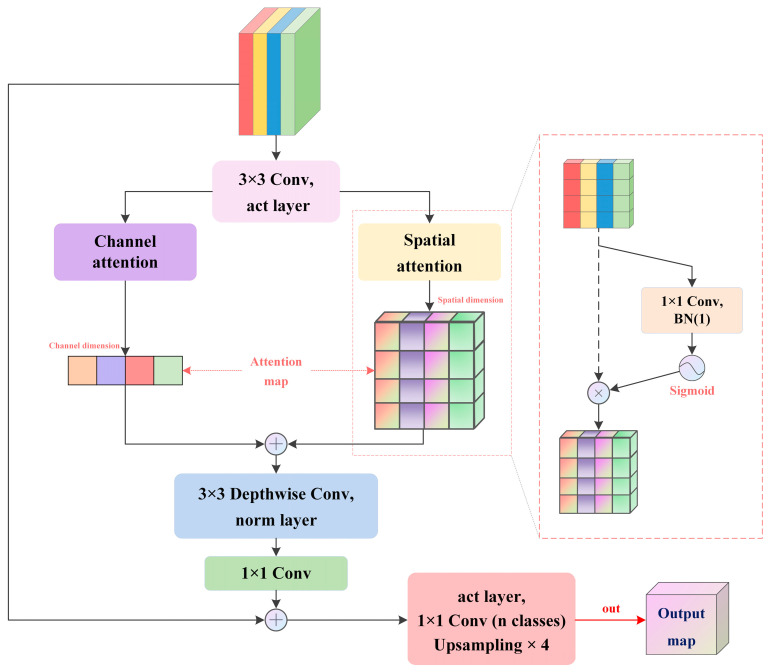
Structure of decoder head.

The overall structure of the proposed feature decoder head is shown in Figure 3. To suppress invalid information before output, we use channel attention and spatial attention to localize the region of interest, respectively. Specifically, in the spatial path, we generate the projection feature map of x ∈ ℝ^B×1×H×W^ by pixel-by-pixel convolution and then adjust its data distribution through the Sigmoid function to generate a spatial attention map with a value range of [0, 1] based on the original feature map. We use the element-wise sum to fuse the attention features of the two paths, then smooth through a 3 × 3 depthwise convolution, and finally perform pointwise convolution for information interaction in the channel dimension, restoring the number of channels. To prevent network degradation, we use residual connections. The process before accessing the classifier can be approximated as

(4) $$\begin{array}{c} x_{\mathrm{out}}=x+{\mathrm{DS}}_{3\times 3}\left(\mathrm{Ch}-A\left({\mathrm{Conv}}_{3\times 3}\left(x\right)\right)+\mathrm{Sp}-A\left({\mathrm{Conv}}_{3\times 3}\left(x\right)\right)\right) \end{array}$$

where Ch-A and Sp-A denote channel attention and spatial attention, respectively.

## 4. Experimental Setup and Results

### 4.1. Dataset and Implementation Details

ZJU-RGB-P (http://www.wangkaiwei.org/dataseteg.html (accessed on 3 December 2023)) is a challenging public dataset of urban scenes, which contains eight foreground classes: building, glass, car, road, tree, sky, pedestrian, and bicycle. Highly reflective objects, such as glass and cars, have RGB information that is easily confused with the environment. In polarized images, highly reflective objects have a specific polarization angle that can be used for better segmentation. The dataset contains data from four polarization angles (triple-band): I_0°_, I_45°_, I_90°_, and I_135°_. Based on the four polarization angle data and the Stokes vector, the polarization secondary products S_0_, S_1_, and S_2_ can be obtained. Among them, S_0_ describes light without any polarization and is also regarded as RGB imagery. The Stokes vector calculation formula is as follows:

(5) $$\begin{array}{c} \vec{S}=\{\begin{array}{c} S_{0}=\\ S_{1}=\\ S_{2}= \end{array}\begin{array}{l} I_{0{}^{\circ}}+I_{45{}^{\circ}}=I_{90{}^{\circ}}+I_{135{}^{\circ}}\\ I_{0{}^{\circ}}-I_{90{}^{\circ}}\\ I_{45{}^{\circ}}-I_{135{}^{\circ}} \end{array} \end{array}$$

Next, the degree of linear polarization (DoLP) and angle of linear polarization (AoLP) of the third-level polarization image products can be further obtained:

(6) $$\begin{array}{c} \mathrm{DoLP}=\frac{\sqrt{{S_{1}}^{2}+{S_{2}}^{2}}}{S_{0}} \end{array}$$

(7) $$\begin{array}{c} \mathrm{AoLP}=\frac{1}{2}\times \arctan\left(\frac{S_{1}}{S_{2}}\right) \end{array}$$

**Figure 4:**
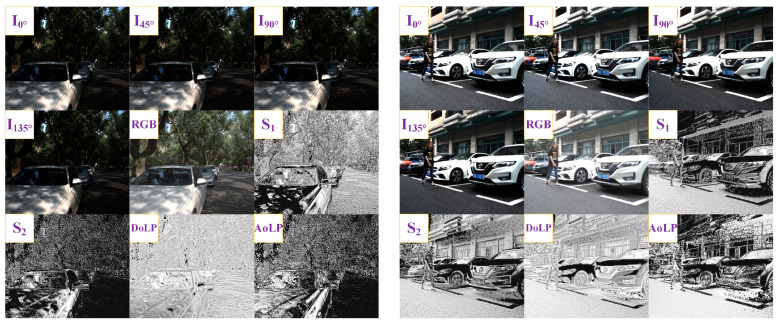
Visualization instances of each modality.

Some visualization instances of each modality in the dataset are shown in Figure 4.

We used an NVIDIA Tesla A100-PCIe GPU (NVIDIA, Santa Clara, CA, USA) with 40 GB memory to deploy experiments. All models use Adam as the optimizer. We took Swin Transformer + UperNet as the baseline model to tune the hyperparameters and, based on the result of normal convergence of this model (no overfitting or underfitting), fine-tuned other models accordingly. The training epoch was set to 100; we used cosine annealing to adjust the learning rate, and a linear learning rate warm-up was followed at the beginning of the training. We cropped the large-resolution images from the dataset into 256 × 256 small-size image blocks. Considering the complex visual representation of the polarized multimodal data, we did not use any data augmentation operations. In this study, the standard accuracy evaluation indicators IoU and mIoU of semantic segmentation were selected to measure the accuracy performance of each model, where IoU can be calculated as

(8) $$\begin{array}{c} \mathrm{IoU}=\frac{\mathrm{TP}}{\mathrm{TP}+\mathrm{FP}+\mathrm{FN}} \end{array}$$

where TP, FP, and FN stand for true positive, false positive, and false negative, respectively, and mIoU is equal to the arithmetic mean of all types of IoU.

### 4.2. Compared Models

We selected some state-of-the-art models to examine the performance of the proposed MixImages, and the selected models include the following categories: (1) unimodal model; (2) multimodal model; (3) model with pure CNN-based backbone; (4) model with pure transformer-based backbone; (5) model with CNN–transformer mixture backbone. The details of each model are shown in Table 2:

### 4.3. Unimodal Models Comparison Results

**Figure 5:**
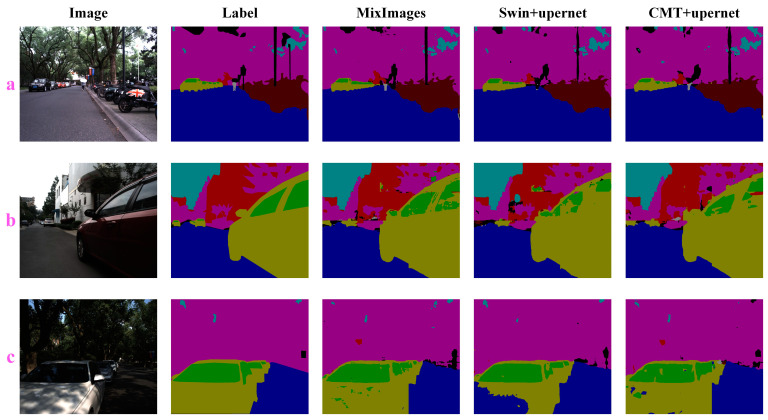
Unimodal model qualitative experiments on the RGB-P dataset. Subfigures (**a**–**c**) show the experimental results under three different samples. The second to fifth columns of the figures show different colors corresponding to different categories: Background (black), Bicycle (brown), Pedestrian (gray), Sky (cyan), Vegetation (magenta), Road (blue), Car (olive green), Glass (green), and Building (red).

In the experiments, we first selected only RGB images as the input of each model to evaluate the performance of MixImages in unimodal benchmarks. The quantitative performance comparison results of the RGB-P dataset are shown in Table 3, and some qualitative performance comparison results are shown in Figure 5 (only the results of the top two models in the control group are given).

RGB-P quantitative comparison results show that our MixImages was consistently superior to the existing technology. Especially compared to Swin + UperNet, which ranks second in mIoU, MixImages achieved a huge improvement of 3.43% mIoU on the basis of similar parameters. The main performance gains are reflected in “glass”, “pedestrian”, and “bicycle”. It is worth noting that in the qualitative comparison results, the Swin + UperNet, with the worst ability to extract detailed features, has a large number of misjudgments (Figure 5c). In natural urban scenes, the shadows mapped on the car surface generate a lot of RGB noise information. Because of the similar color features and blurred boundaries, more texture detail features are needed to better distinguish them. However, the Swin transformer is a pure transformer visual backbone, so the local neighborhood features of the ground objects were not recognized; the basis for the network to make a judgment seems to be only through the color. MixImages and CMT with transformer and CNN mixed backbones avoid this problem well. On the other hand, we believe that another reason for this phenomenon may be due to the use of a 256 × 256 cropping size. For the semantic segmentation model with a pure transformer as the encoder, the effective receptive field of the network is limited to a local range, and the rest is masked, so it cannot provide a basis for judgment through more long-distance dependencies. Although this phenomenon may be alleviated by using a larger crop size, it at least shows that the lack of a CNN as a complementary pure transformer backbone lacks robustness under extreme conditions, while the backbone with mixed operators learns more steady visual representations.

### 4.4. Multimodal Models Comparison Results

**Figure 6:**
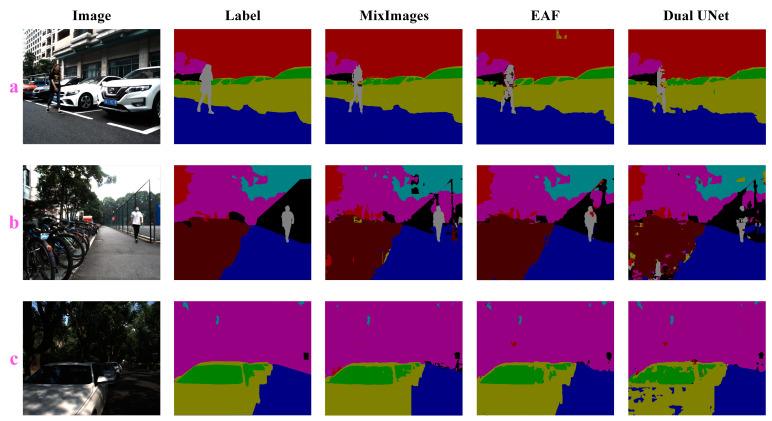
Multimodal model qualitative experiments on the RGB-P dataset. Subfigures **a**–**c** show the experimental results under three different samples. The correspondence between different colors and categories in columns 2–5 of the figures can be found in the caption of Figure 5.

In this part, we evaluate the performance of MixImages in multimodal (dual-modal) benchmarks. Since most multimodal networks can only receive data from two modalities, when RGB is used as the main modal data, only one of the remaining multimodal datasets is selected as additional input. Considering the differences between the two multimodal datasets and referring to existing research, we chose DoLP as the supplementary modal data. Quantitative comparison results are shown in Table 4, and some qualitative performance comparison results are given in Figure 6. The results indicate that MixImages still achieves better accuracy than previous models significantly under this benchmark, e.g., the proposed MixImages maintains an absolute leading position, and its mIoU is significantly better than EAF 4.29%, which is the first in the control group. Also, on the challenging category “pedestrian”, the proposed method outperforms the EAF by 10 points. In addition, according to the visualization results, it is evident that our method is superior.

### 4.5. Performance of MixImages under Different Multimodal Data

In this part, we explore how different combinations of polarization modalities affect the performance of the proposed model. Since the dataset contains as many as nine modalities in total, conducting a complete random permutation of combinations results in an excessively large search space. Therefore, we partitioned the modalities into different image groups (IGs) based on the polarization product levels. Specifically, we use IG_1_ to indicate the input of I_0°_, I_45°_, I_90°_, and I_135°_ together; IG_2_ to indicate the input of RGB (S_0_), S_1_, and S_2_ together; and IG_3_ to indicate the input of DoLP and AoLP together. All the results are shown in Table 5.

The results show that when only one polarization information of DoLP is added, mIoU is improved. However, as the data of different polarization modes were further increased, the accuracy degraded. It was not until the data of all nine modes were fed to the network that the accuracy broke through again. We believe this phenomenon was due to the features of the data. For RGB images, polarization data have not only complementarity but also mutual dissimilarity. For example, for the polarization-sensitive category of “glass”, no matter how the input is changed, the accuracy is always better than using only RGB images (in the qualitative comparison, the feedback is that the salt and pepper noise of the falling shadow car in Figure 5c and Figure 6c has been significantly improved). For the “building” category, the introduction of polarization information blurs the clear visual representation in the RGB image, blindly adding polarization data that are quite different from the RGB visual mode and leading to the IoU always being lower than that of using only RGB data. Until the introduction of I_0°_, I_45°_, I_90°_, and I_135°,_ which are closest to the RGB image, the IoU can be picked up. Therefore, having more polarization multimodal data is not better for urban perception, but the data should be combined with the specific tasks, selecting the appropriate multimodal data as a supplement.

## 5. Conclusions

This work proposes a novel urban perception AI model: MixImages. Different from the previous multimodal studies, we shifted our focus from solely using RGB data to incorporating polarization data to enhance the representation of shadows in urban scenes. We conducted extensive experiments on a public dataset, and the results show that the proposed MixImages can achieve comparable or even better performance than well-designed state-of-the-art semantic segmentation models, whether solely utilizing RGB data or integrating DoLP data. Furthermore, we also explore how different combinations of polarization data affect model performance and find that MixImages can achieve further performance improvement when more data are inputted.

## Figures and Tables

**Table 1:** Hyperparameter settings for different capacity models.

Modal Name	C_1_	D_1, 2, 3, 4_	H′	G′	#Params
MixImages-T	96	(2, 2, 8, 2)	32	8	27M
MixImages-S	96	(2, 2, 24, 2)	32	8	51M
MixImages-M	128	(2, 2, 24, 2)	32	16	90M
MixImages-B	192	(4, 4, 24, 4)	32	16	252M

**Table 2:** Details of the models used for comparison, where “ǂ” indicates multimodal models with double input.

Models	Backbone	Type
MANet (2022) [63]	ResNet-18	CNN-based
TransUNet (2021) [54]	ResNet-50 + ViT-B	CNN-based
UNetFormer (2022) [64]	ResNet-18	CNN-based
CMT + UperNet (2022) [65]	CMT-T	CNN–transformer mixture
Swin Transformer + UperNet (2021) [46]	Swin-T	Transformer-based
MixImages (ours)	MixImages-T	CNN–transformer mixture
ǂ EAF (2021) [57]	ResNet-18	CNN-based
ǂ UisNet (2022) [66]	SegFormer-B3	CNN–transformer mixture
ǂ DIR_DeepLabv3 + (2022) [67]	ResNet-50	CNN-based
ǂ Dual UNet (2022) [68]	UNet	CNN-based
ǂ MixImages (ours)	MixImages-S	CNN–transformer mixture

**Table 3:** Unimodal model quantitative experiments on the RGB-P dataset (%).

Method	Building	Glass	Car	Road	Tree	Sky	Pedestrian	Bicycle	mIoU
MANet	75.96	66.90	83.49	91.56	85.61	75.19	42.58	71.23	74.07
TransUNet	74.01	65.67	81.77	92.79	87.54	75.84	40.48	69.84	73.49
UNetFormer	77.86	64.82	84.35	91.74	88.56	78.32	43.56	70.04	74.91
CMT + UperNet	78.42	65.90	86.49	92.42	90.41	79.62	42.39	72.86	76.06
Swin + UperNet	83.56	67.06	89.06	93.28	91.32	82.95	45.21	75.22	78.46
MixImages	84.26	75.02	91.15	94.31	92.86	85.23	51.07	81.24	81.89

**Table 4:** Multimodal model quantitative experiments on the RGB-P dataset (%).

Method	Building	Glass	Car	Road	Tree	Sky	Pedestrian	Bicycle	mIoU
EAF	82.32	73.12	90.26	88.17	91.06	83.25	53.31	82.46	80.49
UisNet	58.38	42.41	74.75	83.46	87.43	63.75	9.83	46.12	58.27
DIR DeepLabv3+	81.61	74.08	87.54	92.41	90.56	82.43	45.35	83.50	79.69
Dual UNet	82.73	73.01	87.30	93.76	91.03	83.78	48.56	81.71	80.24
MixImages	83.42	78.82	92.69	95.31	93.14	85.86	63.73	85.27	84.78

**Table 5:** Performance of MixImages under different multimodal data input on the RGB-P dataset (%).

Input Data	Building	Glass	Car	Road	Tree	Sky	Pedestrian	Bicycle	mIoU
RGB	84.26	75.02	91.15	94.31	92.86	85.23	51.07	81.24	81.89
RGB + DoLP	83.42	78.82	92.69	95.31	93.14	85.86	63.73	85.27	84.78
RGB + IG_3_	81.78	77.64	91.49	94.88	93.40	87.52	62.41	82.19	83.92
IG_2_ + IG_3_	83.03	78.38	91.51	94.13	93.38	87.71	58.69	80.72	83.44
IG_1_ + IG_2_ + IG_3_	85.64	79.20	93.83	94.26	93.52	87.68	64.02	85.76	85.49

## Data Availability

Data are contained within the article.
